# Challenge of Naïve and Vaccinated Pigs with a Vaccine-Derived Recombinant Porcine Reproductive and Respiratory Syndrome Virus 1 Strain (Horsens Strain)

**DOI:** 10.3390/vaccines9050417

**Published:** 2021-04-22

**Authors:** Lise K. Kvisgaard, Lars E. Larsen, Charlotte S. Kristensen, Frédéric Paboeuf, Patricia Renson, Olivier Bourry

**Affiliations:** 1 Institute for Veterinary and Animal Sciences, Section for Veterinary Clinical Microbiology, University of Copenhagen, 1870 Frederiksberg C, Denmark; lael@sund.ku.dk; 2 Danish Pig Research Centre, SEGES, 8200 Aarhus N, Denmark; csk@seges.dk; 3 Laboratoire de Ploufragan-Plouzané-Niort, Agence Nationale de Sécurité Sanitaire de l’Alimentation, de l’Environnement et du Travail (Anses), 22440 Ploufragan, France; frederic.paboeuf@anses.fr (F.P.); Patricia.RENSON@anses.fr (P.R.); Olivier.BOURRY@anses.fr (O.B.)

**Keywords:** porcine reproductive and respiratory syndrome virus, recombination, vaccines, swine, PRRSV, vaccine efficacy

## Abstract

In July 2019, a vaccine-derived recombinant Porcine reproductive and respiratory syndrome virus 1 strain (PRRSV-1) (Horsens strain) infected more than 40 Danish sow herds, resulting in severe losses. In the present study, the pathogenicity of the recombinant Horsens strain was assessed and compared to a reference PRRSV-1 strain using a well-characterized experimental model in young SPF pigs. Furthermore, the efficacies of three different PRRSV-1 MLV vaccines to protect pigs against challenge with the recombinant strain were assessed. Following challenge, the unvaccinated pigs challenged with the Horsens strain had significant increased viral load in serum compared to all other groups. No macroscopic changes were observed at necropsy, but tissue from the lungs and tonsils from almost all pigs were PRRSV-positive. The viral load in serum was lower in all vaccinated groups compared to the unvaccinated group challenged with the Horsens strain, and only small differences were seen among the vaccinated groups. The findings in the present study, combined with two other recent reports, indicate that this recombinant “Horsens” strain indeed is capable of inducing infection in growing pigs as well as in pregnant sows that is comparable to or even exceeding those induced by typical PRRSV-1, subtype 1 strains. However, absence of notable clinical signs and lack of significant macroscopic changes indicate that this strain is less virulent than previously characterized highly virulent PRRSV-1 strains.

## 1. Introduction

Porcine reproductive and respiratory syndrome virus (PRRSV) has, since its first appearance in the beginning of the 1990s, been one of the major health challenges in commercial pig production. Two species of PRRSV, termed PRRSV-1 and PRRSV-2, which share only 60% identity on the genetic level, are circulating globally [1]. There is a variety of PRRSV programs in place for the control of PRRSV in different herds, but PRRSV is often circulating in the nursery and among growers/fatteners. Regularly, mass sow vaccinations with Modified Live Virus (MLV) vaccines are used in many herds, whereas piglet vaccination is less commonly used. 

In July 2019, a PRRSV-1 virus was detected in samples taken as part of the routine PRRSV surveillance in one of the Danish PRRSV-negative boar stations. Full genome sequencing revealed that the strain was resulting from a recombination between the Amervac strain (Unistrain PRRS vaccine; Hipra) and the 96V198 strain (Suvaxyn PRRS; Zoetis AH) [2]. The major parent was the 96V198 strain that spanned ORFs 1–2 and part of ORF 3 (83.5% of the genome), and the minor parent was the Amervac strain, which constituted the remaining part of the genome (16.5% of the genome). More than 700 herds received potentially contaminated semen, and infection with this strain was confirmed by sequencing from at least 38 of these herds. Due to the geographical origin of the recombinant strain, it was named the ‘Horsens strain’.

Follow-up in 13 of the affected herds revealed production losses twice of what is normally observed in Danish herds infected with PRRSV-1 [3]. The clinical signs included sustained reproductive failures and high piglet mortality. Furthermore, the load of virus found in processing fluids, lungs and serum of infected pigs exceeded the levels normally seen in samples from PRRSV-1 diseased pigs, which indicate a very high level of viral replication. Compiled, the available data indicate that this strain has regained a profound level of virulence despite that the two parent viruses are attenuated vaccine strains. Assessment of the pathogenicity of PRRSV strains under field conditions is, however, difficult because disease severity is determined by a wide range of other contributing factors in the herd, emphasizing that proper evaluation of the pathogenicity of PRRSV strains requires controlled experimental trials. Recently, an experimental study performed in sows with the Horsens strain showed that the virus induced reproductive failures and high levels of viremia in pregnant sows, but to a lesser extent than the field strain Olot/91 [4].

The aims of the present study were to compare the pathogenicity of the recombinant Horsens strain with a typical PRRSV-1 field strain and to investigate the efficacy of three different commercial PRRSV-1 MLV vaccines to protect pigs against challenge with the recombinant strain using a well-characterized experimental model in young specific pathogen-free (SPF) pigs.

## 2. Material and Methods

### 2.1. Experimental Design

In total, 36 four-week-old PRRSV-negative purebred large-white pigs of both sexes were included in the study. The pigs were from the SPF pig herd of Anses Ploufragan Laboratory, free of a range of pathogens including PRRSV, Porcine circovirus type 2 (PCV2) virus, swine influenza A virus, *Actinobacillus pleuropneumoniae* (AP) and *Mycoplasma hyopneumoniae*. The pigs were housed at the experimental animal facilities at Anses Ploufragan Laboratory under appropriate biosecurity conditions. After weaning, the pigs were allocated into six groups of six pigs according to gender equality, mean body weight and even distribution of litter origin. The groups were housed in separate rooms with three pigs per pen. The pigs were identified by ear tags. They were fed ad libitum with a post-weaning diet (during the first 40 days after weaning) and then with a growing-finishing diet until the end of the study (no zinc supplementation in these 2 diets). They had access to water ad libitum and to manipulable tools.

Four days after arrival (0 days post-vaccination, dpv 0), the pigs in groups 1–3 were vaccinated by the IM route with either Suvaxyn PRRS MLV (SUV) (Zoetis, Zaventem, Belgium), Unistrain PRRS (UNI) (HIPRA, Amer, Girona, Spain) or Porcilis PRRS (PORCI) (MSD, Beaucouzé, France), according to the summary of product characteristics (SPC) of each vaccine. The pigs in groups 4–6 were left unvaccinated (Table 1). 

Four weeks after the vaccination (dpv 28), all pigs in groups 1–4 were challenged intranasally with 5 mL of cell culture medium containing a total of 5 × 10^5^ TCID50/pig of the recombinant “Horsens” strain (REC). The pigs in group 5 were challenged with 5 × 10^5^ TCID50/pig of the reference PRRSV-1 strain “Finistere” (REF). The pigs in group 6 were left unchallenged (Table 1).

The study was carried out in accordance with the French legislation on animal experiments. The study protocol was approved by animal welfare committee No. 16 (approval No. 2020-02-04-09) on 5 February 2020 and by the research ministry (No. APAFiS 2020012315156912) on 14 February 2020.

### 2.2. Challenge Viruses and Inoculum

The “Horsens” inoculum contained 10^5^ culture infective dose per mL (TCID_50_/mL) of PRRSV titrated in PAMs (porcine alveolar macrophages) and suspended in RPMI medium (passage 4 amplified on MARC-145 cells.) The “Finistere” inoculum contained 10^5^ TCID_50_/mL of PRRSV titrated in PAMs and suspended in RPMI medium (passage 3 amplified in PAMs).

### 2.3. Genetic Comparisons of the Vaccine and Challenge Strains 

Strains included in the genetic analyses included the strains of the vaccines Porcilis PRRS (Strain DV; accession number KJ127878), Unistrain PRRS (Strain Amervac; accession number GU067771), Suvaxyn PRRS (Strain 96V198; accession number MK876228) and the challenge strains Horsens (accession number MN603982) and Finistere (PRRS-FR-2005-29-24-1; accession number KY366411).

A phylogenetic tree was constructed based on the ORF1–ORF7 nucleotide sequences of the PRRSV strains utilized in this study together with global representative PRRSV-1 strains. The alignment was performed with MUSCLE (Multiple Sequence Comparison by Log-Expectation), and the phylogenetic tree was constructed with the Neighbor-Joining algorithm and 1000 bootstrap replicates and visualized with FigTree v.1.4.4., as previously described [2]. Pairwise amino acid comparison of the three vaccine strains to the challenge strain (Horsens) and reference strain (Finistere) was performed based on an amino acid alignment of the GP5 protein.

### 2.4. Sampling

Blood samples (9 mL plain venoject tubes) were collected on days 3, 7, 14, 24, 31, 36, 42, 49 and 56 after vaccination (dpv). Oral fluid (OF) samples were collected on the same time points (one rope per pen (Idexx Oral Fluid Collection Kit)) and stored at −80 °C until testing. Serum was separated from the blood by centrifugation and stored at −80 °C until testing. 

### 2.5. Clinical Observation

Clinical score was monitored daily using a scoring grid as previously described [5]. Rectal temperature was recorded on dpv 0–4, 7–8, 11, 14, 18, 21, 24, 28–38, 42, 44, 49, 51–52 and 56. 

The pigs were weighed at the start of the study and then weekly until necropsy. 

### 2.6. Necropsy

The pigs were anesthetized (Zoletil, Virbac, Carros, 10 mg/kg IM) and then euthanized by exsanguination between 35 and 39 days after challenge, according to Table 1. Complete necropsies were performed with scoring of lung lesions. Tissue samples of the lung Diaphragmatic Right Lobe (DRL), lung Diaphragmatic Left Lobe (DLL), lung Apical Right Lobe (ARL), tonsils and tracheobronchial lymph nodes (TB LN) were collected for PRRSV viral load quantification and stored at −80 °C until use. 

### 2.7. RNA Extraction and RT-qPCR Assays

Total RNA was extracted from serum samples using the QIAcube HT robot (QIAGEN) and the Cador Pathogen 96 QIAcube HT kit (INDICAL Bioscience). Total RNA was extracted from 200 µL aliquots utilizing the purification protocol: ‘Cador Pathogen 96 QIACube HT V3’. RNA was eluted in 120 µL. Total RNA from oral fluids samples were extracted from 140 µL aliquots with the QIAamp^®^ Viral RNA Mini Kit (QIAGEN) using the QIAGEN QIAcube Connect extraction robot and protocol: ‘Purification of viral RNA from cell-free body fluids’. RNA was eluted in 60 µL. Prior to the extraction of total RNA from oral fluids, the samples were added to a metal bead and homogenized on a Tissuelyser II at 30 Hz, 15 s, following centrifugation for 3 min at 5500× *g*. Total RNA from tonsils, tracheobronchial lymph nodes and lung tissue samples were extracted from homogenate supernatants as previously described [6]. Total RNA was extracted from 600 µL tissue homogenate using the QIAcube Connect extraction robot with the RNeasy Mini kit (QIAGEN) and protocol: ‘Purification of total RNA from animal tissues’. RNA was eluted in 60 µL. Known PRRSV-positive samples and negative controls were included in each extraction. Extracted RNA was stored at −80 °C.

For detection of PRRSV-1, the previously published RT-qPCR “Kleiboeker mod-1” primers and probes targeting ORF6 and ORF7 were used [7].

A 10-fold dilution series of extracted RNA from the recombinant Horsens and the Finistere cell culture isolates gave similar results and was used for the construction of a standard curve. The 10^4^ RNA dilution corresponding to 1.2 × 10^3^ Relative Equivalent Units (REUs) (Ct-value: 28.54) was subsequently included in each RT-qPCR run. The obtained Ct-values from the screened samples were converted into REUs by importing the standard curve to the Rotor-Gene Q Series Software v2.3.1 (QIAGEN, Hilden, Germany), setting the 10^4^-dilution sample as ‘standard type’ with the given concentration of 1.2 × 10^3^.

#### SYBR Green Real-Time RT-PCR Assay Specific for the Horsens Strain

A SYBR green real-time RT-PCR assay specific for the Horsens strain was designed. The primers were designed to target ORF3, spanning the recombination breakpoint resulting in a 251 bp amplicon. The real-time RT-PCR was run on a Rotor-Gene Q machine (QIAGEN) in a total volume of 25 µL using the Qiagen OneStep RT-PCR kit with 2 µL extracted RNA, 1.5 µM of each primer and 2× SYBR green (SYBR Green I Nucleic Acid Gel Stain 10,000×, CAMBREX) final concentrations. The amplification profile was: 50 °C, 30 min; 95 °C, 15 min; 45 cycles (94 °C, 15 s; 60 °C, 15 s; 72 °C, 60 s); 72 °C, 5 min; 50 °C, 1 min, immediately followed by a melting point analysis, ramping from 50 to 99 °C, rising 1 °C per step, holding 5 s at each temperature increase. The fluorescence signal was measured at 72 °C during each PCR cycle and at each temperature increase during melting analysis. The fluorescent signals were analyzed with Rotor-Gene Q software v. 2.3.1 (QIAGEN), setting the NTC threshold at 10% and cycle threshold at 0.01. 

### 2.8. Antibody ELISA

Serum samples were screened for PRRSV antibodies using the commercial kits IDEXX PRRS X3 Ab test, according to the protocol supplied by the manufacturer.

### 2.9. Laboratory Analyses

All serum and OF samples collected at days 3, 8, 14, 21 and 28 post-challenge (dpc) were tested in real-time RT-PCR for PRRS virus, and serum was tested in ELISA for PRRSV antibodies. Samples from the serum from dpv 24 (prior to challenge) and 36 (8 dpc) were tested by the Horsens-specific real-time RT-PCR assay.

The tissue samples (lung, tonsils, tracheobronchic lymph nodes) collected from each pig at necropsy were tested by the general real-time RT-PCR. 

### 2.10. Statistical Analysis

All the data and calculated areas under the curve (AUC) were compared between groups using the Kruskal–Wallis test, followed by Holm’s corrected Wilcoxon’s pairwise comparisons, using R software version 4.0.2. A value of *p* < 0.05 was considered significant. In figures, different letters indicate the significant differences obtained by comparing one group either to the SUV group (a), to the UNI group (b), to the PORCI group (c), or to the REF group (d).

## 3. Results

### 3.1. Genetic and Antigenic Analysis of Vaccine and Challenge Strains

**Figure 1:**
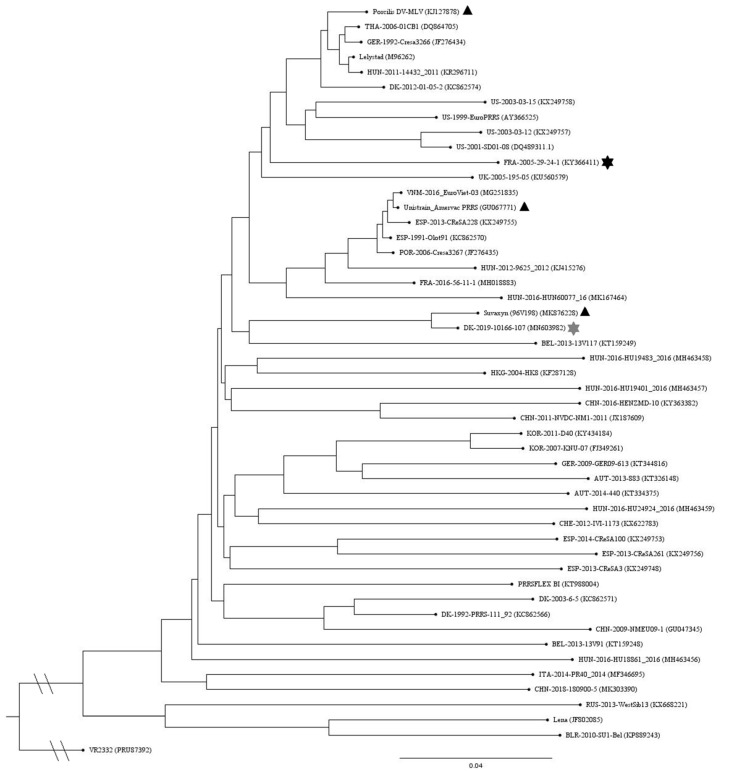
Phylogenetic tree of ORF1–ORF7 global representative PRRSV-1 nucleotide sequences. The three vaccine strains are marked with black triangles and the challenge (REC) and reference (REF) strains are marked with grey and black stars, respectively. The tree was constructed with the Neighbor-Joining algorithm and 1000 bootstrap replicates and visualized in FigTree v.1.4.4.

A phylogenetic tree of ORF1–ORF7 global representative PRRSV-1 nucleotide sequences is shown in Figure 1. The REC strain has the shortest distance to the Suvaxyn vaccine strain. Pairwise comparison of amino acid sequences of GP5 (ORF5) of the REC strain to the three vaccine strains shows the highest similarity to the Unistrain vaccine strain, with 97.51% identity, and only 91% similarity to the Suvaxyn and Porcilis vaccine strains, respectively (data not shown).

### 3.2. Clinical Signs, Rectal Temperature and Body Weight Gains 

No respiratory clinical symptoms were observed in any pig after vaccination or after challenge. During the first week after challenge, the rectal temperature in the REC group exceeded those of the other groups, however, using a threshold of 40.0 °C, only very few pigs developed fever during the post-challenge period (data not shown). 

**Figure 2:**
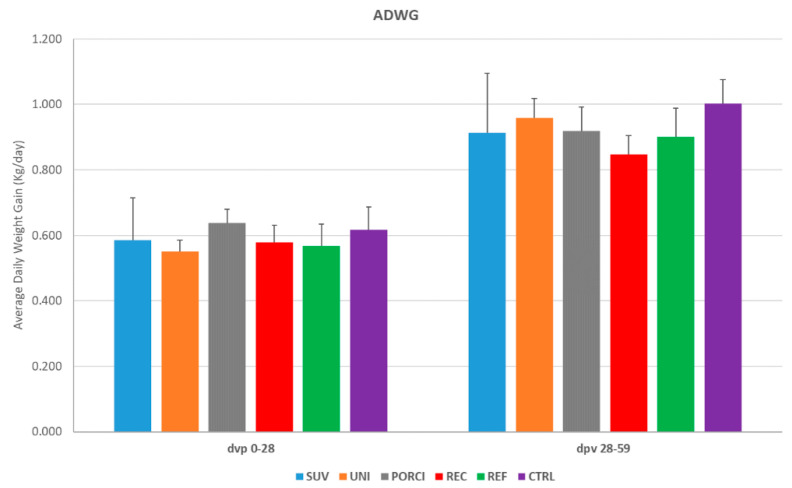
Average daily body weight gain (ADWG) (kg per day) from start to finish of the study at the group level. The pigs in the SUV, UNI and PORCI groups were vaccinated with the vaccine Suvaxyn PRRS, Unistrain or Porcilis PRRS at day 0 post-vaccination (dpv) and challenged at dpv 28 with the recombinant Horsens strain together with an unvaccinated group of pigs (REC). Another group of unvaccinated pigs was challenged on dpv 28 with a reference strain (REF). The control group (CTRL) was left unvaccinated and unchallenged.

The average daily body weight gain (ADWG, kg per day) for the post-vaccination (dpv 0–28) and post-challenge (dpv 28–59) periods on the group level are shown in Figure 2 and the data for each study week are shown in Figure S1. Overall, there were no significant differences between the ADWG of the groups; however, the REC group had a significantly decreased ADWG during the first week after challenge when compared to the UNI group only. 

### 3.3. Macroscopic Changes

At necropsy, slightly enlarged and congestive lymph nodes were observed for most of the infected pigs. No other lung lesions were observed in any of the pigs. 

### 3.4. PRRS Virus in Serum and OF

**Figure 3:**
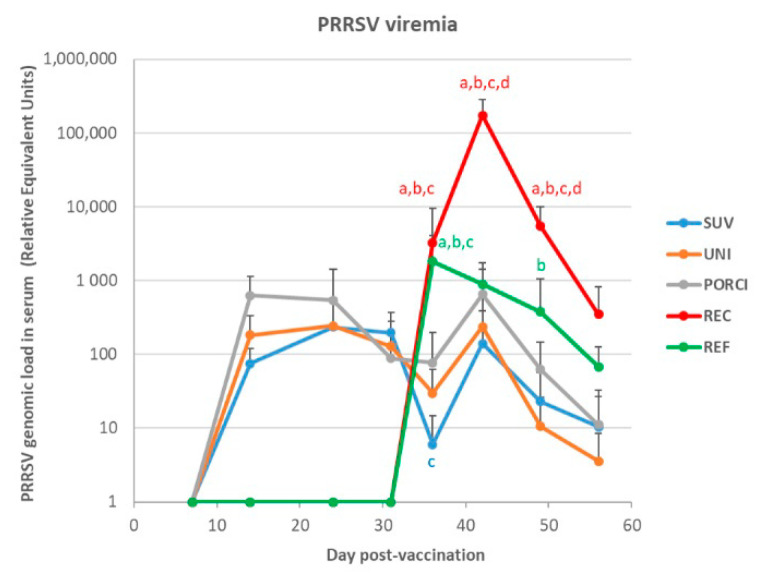
Results of the RT-qPCR analyses of serum samples following vaccination and challenge expressed as relative equivalent units (REUs). The pigs in the SUV, UNI and PORCI groups were vaccinated with the vaccine Suvaxyn PRRS, Unistrain or Porcilis PRRS at day 0 post-vaccination (dpv) and challenged at dpv 28 with the recombinant Horsens strain together with an unvaccinated group of pigs (REC). Another group of unvaccinated pigs was challenged on dpv 28 with a reference strain (REF). Different letters indicate significant difference obtained comparing the considered group to the SUV group (a), the UNI group (b), the PORCI group (c) or the REF group (d), with *p* < 0.05.

Results of the RT-qPCR analyses of serum samples following vaccination and challenge for all of the pigs challenged at dpv 28 are shown in Figure 3. None of the samples from the unvaccinated and unchallenged control group tested positive at any sampling point. At dpv 7, viral RNA was detected in all vaccinated animals with relative equivalent units (REUs) between 11 and 1380. The PORCI group peaked at dpv 7, whereas the SUV and UNI groups peaked at dpv 14, followed by a steady decrease until dpv 31. No viral RNA was detected in any of the unvaccinated groups prior to challenge. Following challenge, the REUs of the vaccinated groups increased between dpv 31 and dpv 36 followed by a steady decrease until termination of the study. The group of pigs challenged with the reference strain (REF) and the recombinant strain (REC) had a marked increase in REUs at dpv 31 (dpc 3) followed by a steady decrease in REUs of the REF strain. In contrast, the REUs of the pigs challenged with the REC strain increased to >100,000 at dpv 36 followed by a marked decrease until termination of the study. There was a significantly higher viral load in serum in the REC group compared to all the vaccinated groups at dpv 31 (dpc 3) and to all groups at dpv 36 (dpc 8) and dpv 42 (14 dpc). Similarly, there was a significant difference between the REF and all the vaccinated groups at dpv 31 and between the REF and the UNI groups at dpv 42. The only significant difference among the vaccinated groups was a significantly higher viral load at dpv 31 in the PORCI group compared to the SUV group.

**Figure 4:**
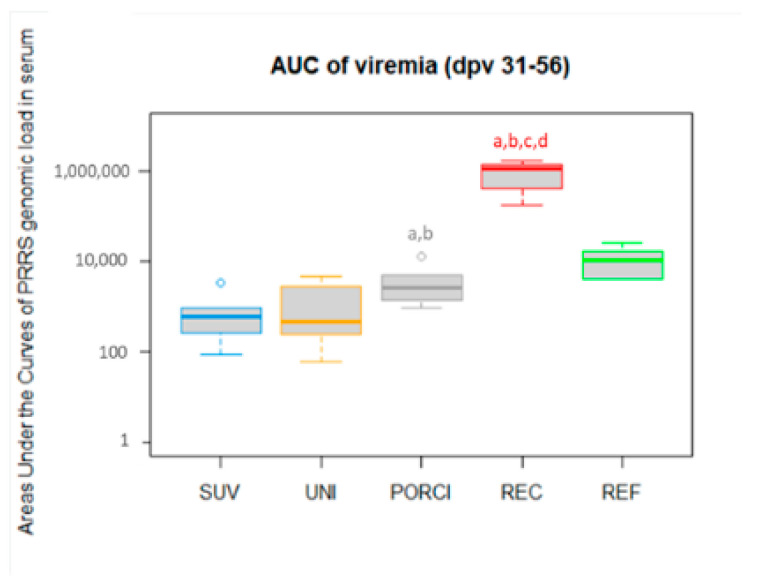
The individual areas under the curves (AUC) of viral loads quantified in serum from dpv 31 to 56 (post-challenge period). The SUV, UNI and PORCI groups were vaccinated with the vaccines Suvaxyn, Unistrain or Porcilis PRRS at day 0, and challenged at dpv 28. The REC and REF groups were unvaccinated. Different letters indicate significant difference obtained comparing the considered group to the SUV group (a), the UNI group (b), the PORCI group (c) or the REF group (d), with *p* < 0.05.

The total viral load after challenge (represented by the area under the curve for the viremia) in all the groups challenged either with the REC or the REF strain are shown in Figure 4. The REC group had a significantly higher viral load compared to all the vaccinated groups and to the REF group. Among the vaccinated groups challenged with the REC strain, the total viral load was significantly higher in the PORCI group compared to the UNI and SUV groups.

Tests of samples from dpv 24 with the RT-PCR assay specific for the REC strain revealed negative results, whereas tests of serum samples from dpv 36 (dpc 8) generated positive results (data not shown). These results confirmed that the vaccinated animals indeed were infected by the REC strain after challenge.

Oral fluids (OF) were collected at dpv 24 (dpc −4) and dpc 3, and subsequently, every week after challenge until the end of the study (dpc 8, 14, 21 and 28) (Table 2). PRRSV-1 was detected in one pen of each vaccinated group at dpc –4. After challenge, the PORCI group had at least one pen positive for PRRSV-1 until dpc 21, where all OF samples in all three vaccinated groups tested negative for PRRSV-1. At dpc 28, one pen of all three vaccine groups tested positive. The REC and REF groups tested positive in both pens at dpc 3 and 8, but only the REC group stayed positive in both pens until dpc 21. At necropsy at dpc 28, all OF samples were negative in the REC and REF groups. 

### 3.5. PRRS Viral Load in Tissue

**Figure 5:**
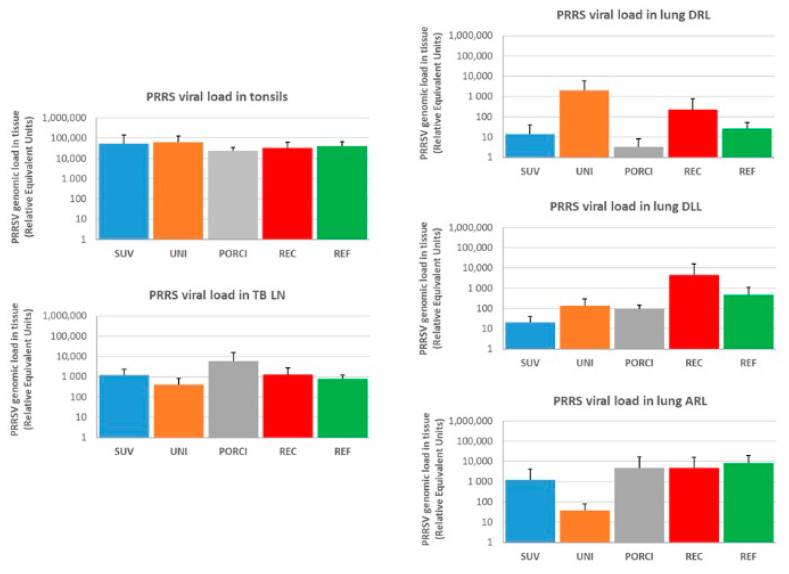
Viral load measured by qRT-PCR (REU) in the lung Diaphragmatic Right lobe (DRL), lung Diaphragmatic Left Lobe (DLL), lung Apical Right lobe (ARL), tonsils and tracheobronchial lymph nodes (TB LN). The SUV, UNI and PORCI groups were vaccinated with the vaccines Suvaxyn PRRS, Unistrain or Porcilis PRRS at dpv 0 and challenged with REC at dpv 28. The REC and REF groups were unvaccinated and challenged with the REC or REF strains at dpv 28.

The viral load in the lung Diaphragmatic Right Lobe (DRL), lung Diaphragmatic Left Lobe (DLL), lung Apical Right Lobe (ARL), tonsils and tracheobronchial lymph nodes (TB LN) are presented in Figure 5. None of the tissue samples from the control animals (CTRL) tested positive. The highest viral loads in the other groups were detected in the tonsil (10^4^–10^5^ REUs) followed by the lymph node (approximately 10^3^ REUs). The load in the different lung lobes varied between the groups and animals but were in general between 10 and 10^3^ REUs. There were no significant differences in viral loads in any of the tested tissues among the challenged groups.

### 3.6. PRRSV Antibodies

The development of antibodies after vaccination and challenge are shown in Figure S2. Antibodies against PRRSV could be detected in all vaccinated groups at dpv 14 and remain at a high level until the termination of the study. Antibodies were detected in the REC and REF groups 8 days after challenge (dpv 36) and increased between 8 and 14 days after challenge and remained at a high level until termination of the study. A significant difference in the level of antibodies was seen in the REC or the REF group compared to all of the vaccinated groups at 36 dpv. In addition, significantly higher levels of antibodies were detected in the PORCI group compared to the SUV group at dpv 36, 49 and 56.

## 4. Discussion

The aims of the present study were to compare the pathogenicity of the recombinant Horsens strain (REC) with a typical PRRSV-1 strain (REF) and to investigate the efficacy of three different commercial PRRSV-1 MLV vaccines to protect pigs against challenge with the recombinant strain using a well-characterized experimental model in young SFP pigs [8]. Apart from a transient rise in rectal temperature in the REC group, few clinical signs were observed after challenge. The average daily body weight gain was lower for the REC group compared to the other groups during the first week after challenge, but the difference was only statistically significant between the REC and the UNI groups. In general, it is difficult to reproduce severe clinical signs seen under field conditions in young SPF pigs in experimental trials with European field isolates of PRRSV-1 [9]. Instead, the magnitude and duration of virus load in serum are often used as a correlate of viral virulence [10,11,12] and protective effects of vaccination [8,13,14,15]. In the present study, the total viral load in serum of unvaccinated pigs infected with the “Horsens” strain were significantly higher compared to pigs infected with the reference strain “Finistere”, with the maximum virus titer in serum 100-fold higher in the pigs infected with the “Horsens” strain. This finding is in accordance with other studies on so-called high-virulent or “hot” PRRSV-1 strains, such as the East-European PRRSV-1, subtype-3 strain “Lena” [16], the subtype-2 strain “Bor” [10] and the subtype-1 strain “Acro” [17]. In contrast to these studies, there were only limited differences in clinical signs, viral load in tissue and degree of lung lesions in our study, which may indicate that the “Horsens” strain is less virulent than these virus strains. Differences in the outcome of experimental trials may also be due to differences in experimental designs, however, a previous study with the “Lena” strain performed using the same protocol, the same animal facility and pigs from the same source indeed were capable of inducing severe clinical signs [18]. This indicates that the lack of severe clinical disease in the present study was not related to the experimental set-up. Furthermore, the pigs were killed four weeks after challenge, which makes evaluation of the pathological changes inconclusive. The experience in the field was that the Horsens strain behaved more aggressively than typical European PRRSV-1 strains and therefore, we wanted to compare the Horsens strain to a typical PRRSV-1 strain, such as Finistere.

All three vaccines included in the present study had a significant impact on the viral load in serum after challenge with the “Horsens” strain. The group vaccinated with the Porcilis vaccine had a significantly higher total viral load compared to the two other vaccinated groups after challenge. The Unistrain vaccine strain—Amervac—is 97.3% identical (amino acid level) to the “Horsens” strain in GP5, whereas the vaccine strains in Porcilis (DV) and Suvaxyn (96V198) are only 91.5% and 91.1% identical to the challenge strain. Overall, these data indicate that PRRSV outbreaks caused by the “Horsens” strain may be efficiently controlled by MLV vaccines containing a heterologous strain, but there may be differences in the level of protection between vaccines. It should be noticed that some of the herds that experienced outbreaks with the Horsens strain in 2019 managed to control the outbreak by employing mass-vaccination also with the Porcilis PRRS vaccine [3].

Recombination between PRRSV field strains has been extensively described for PRRSV-2 [19,20] and less frequently for PRRSV-1 [21,22]. More rarely, recombination between PRRSV-1 field strains and PRRSV-1 MLV vaccine strains has been reported [23,24]. Recombination between two PRRSV-1 vaccine strains has only been reported once from France, where a recombinant between the “DV” vaccine strain (Porcilis PRRS, MSD) and the “Amervac” strain (Unistrain, Hipra) was detected in a single herd [25]. For some of the PRRSV-2 recombinant viruses, there seems to be an increased virulence of the recombinant strains compared to their parental strains, whereas others do not seem to increase the virulence [26]. Nevertheless, an experimental study revealed that the Porcilis PRRS/Unistrain recombinant virus mentioned above seemed to be more virulent than the two parent PRRSV-1 vaccine viruses when given intramuscularly [15].

A recent paper on the “Horsens” recombinant strain revealed losses in infected herds exceeding those normally seen in Danish herds in connection to PRRSV-1 outbreaks [3]. In addition, infection of pregnant sows in the last trimester induced severe reproductive failures, however, to a lesser extent than those seen following another well-characterized PRRSV-1 strain (Olot91) [4].

## 5. Conclusions

The findings in the present study, combined with two recent studies, indicate that this recombinant “Horsens” strain indeed is capable of inducing infection in pigs as well as in pregnant sows, that is comparable to, or even exceeding those induced by typical PRRSV-1, subtype 1 strains. The viral load measured in serum was comparable to viral loads induced by high virulent PRRSV-1 strains, however, absence of notable clinical signs, lack of fever and lack of significant macroscopic changes indicate that this strain is less virulent than East-European PRRSV-1, subtype 2 and 3 strains, such as “Bor” and “Lena”, and the PRRSV-1 subtype 1 strain “Acro”. Further studies are needed to assess if this strain is more transmissible under field conditions.

## Figures and Tables

**Table 1:** Experimental design and groups. The pigs in groups 1–3 were vaccinated with either Suvaxyn PRRS MLV (SUV), Unistrain PRRS (UNI) or Porcilis PRRS (PORCI), according to the SPC of each vaccine. The pigs in groups 4–6 were left unvaccinated. At dpv 28, all pigs in groups 1–4 were challenged with the recombinant “Horsens” strain (REC) and the pigs in group 5 were challenged with the “Finistere” strain (REF).

Group	No. of Pigs	Vaccination (dpv 0)	Challenge (dpv 28)
1 (SUV)	6	Suvaxyn PRRS MLV	Horsens
2 (UNI)	6	Unistrain^®^PRRS	Horsens
3 (PORCI)	6	Porcilis^®^PRRS	Horsens
4 (REC)	6	None	Horsens
5 (REF)	6	None	Finistere
6 (CTRL)	6	None	None

**Table 2:** Results of qRT-PCR test of oral fluid (OF) samples before (−4) and at day 3, 8, 14, 21 and 28 post-challenge. Negative samples are indicated as blank, one out of two positives in grey and two out of two OF positives in black.

	Days Post-Challenge					
Group	−4	3	8	14	21	28
SUV						
UNI						
PORCI						
REC						
REF						

## Data Availability

The raw data supporting the conclusions of this manuscript will be made available by the authors, without undue reservation, to any qualified researcher.

## Supplementary Materials

The following are available online at https://www.mdpi.com/article/10.3390/vaccines9050417/s1, Figure S1: Average daily body weight gain (ADWG) from week to week. Figure S2: PRRSV antibodies in serum after vaccination and challenge expressed as OD ratios.
