# Supplementary material for: Challenge of Naïve and Vaccinated Pigs with a Vaccine-Derived Recombinant Porcine Reproductive and Respiratory Syndrome Virus 1 Strain (Horsens Strain)
Source: Vaccines (Basel). 2021 Apr 22;9(5):417. doi: 10.3390/vaccines9050417 (PMC8143564; doi:10.3390/vaccines9050417)
Supplement: Supplementary file 1 [file vaccines-09-00417-s001.zip › vaccines-1176453-supplementary.pdf]

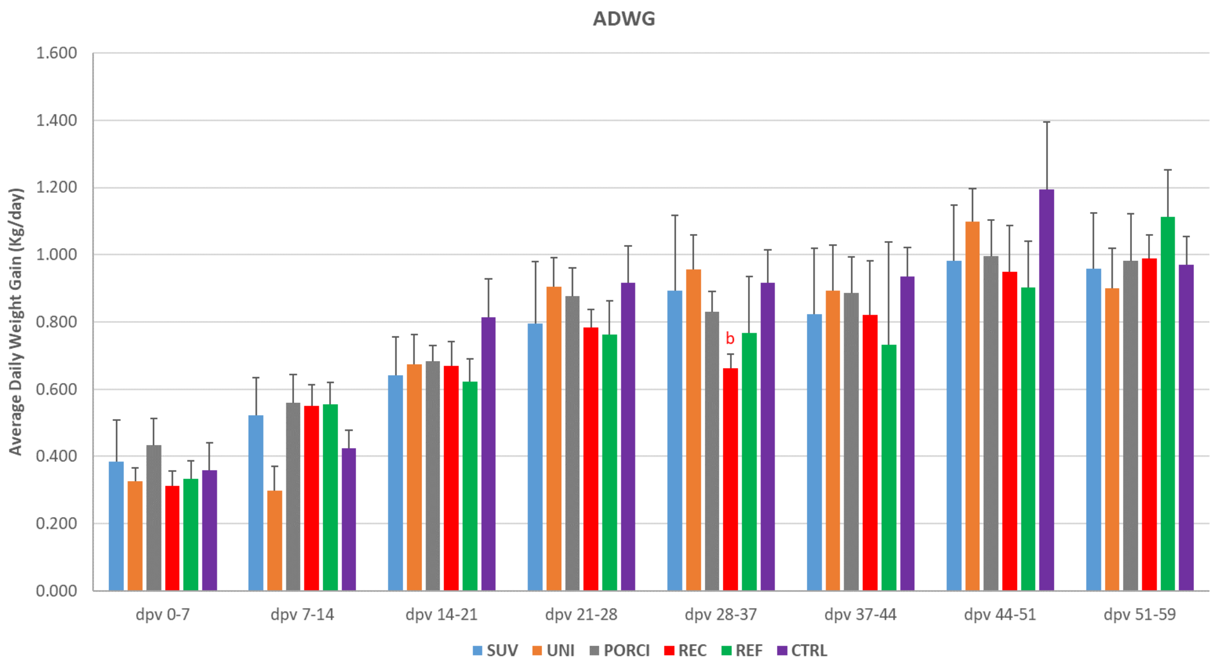


**Figure 1.** Average daily body weight gain (ADWG) from week to week (kg per day). The pigs in the SUV, UNI and PORCI group were vaccinated with the vaccine Suvaxyn PRRS, Unistrain or Porcilis PRRS at day 0 post-vaccination (dpv) and challenged at dpv 28 with the recombinant Horsens strain together with an unvaccinated group of pigs (REC). Another group of unvaccinated pigs was challenged on dpv 28 with a reference strain (REF). The control group (CTRL) was left unvaccinated and unchallenged. Different letter indicates significant difference obtained comparing the considered group to the SUV group (a), the UNI group (b), the PORCI group (c) or the REF group (d) with $p < 0.05$.

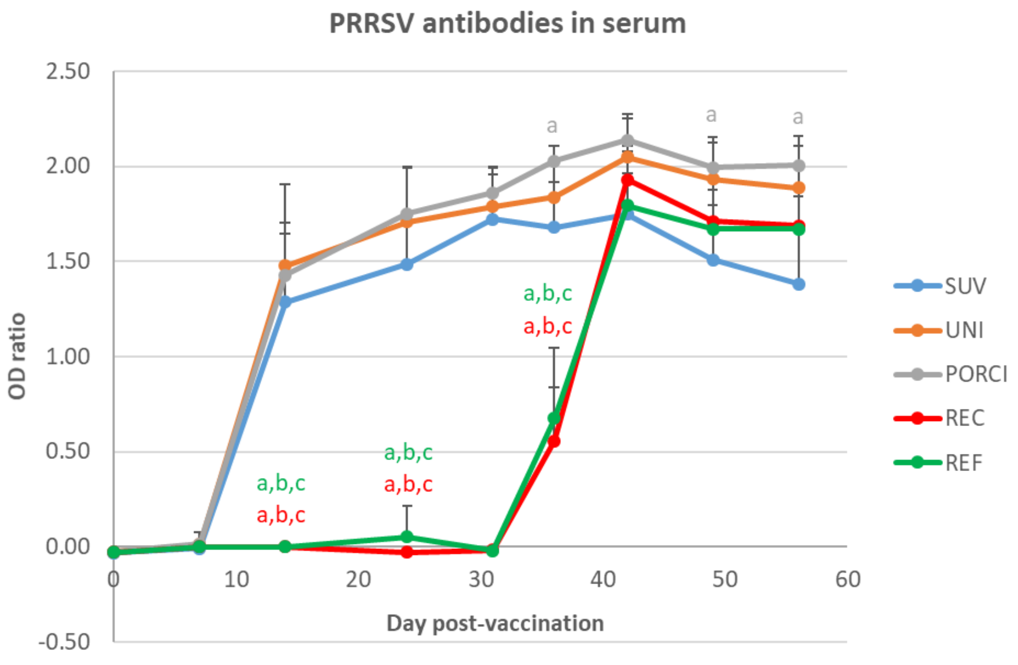


**Figure 2.** ELISA results expressed as OD ratio after vaccination and challenge. The pigs in the SUV, UNI and PORCI group were vaccinated with the vaccine Suvaxyn PRRS, Unistrain or Porcilis PRRS at day 0 post-vaccination (dpv) and challenged at dpv 28 with the recombinant Horsens strain together with an unvaccinated group of pigs (REC). Another group of unvaccinated pigs was challenged on dpv 28 with a reference strain (REF). The control group (CTRL) was left unvaccinated and unchallenged. Different letter indicates significant difference obtained comparing the considered group to the SUV group (a), the UNI group (b), the PORCI group (c) or the REF group (d) with $p < 0.05$.
